# Research on Modeling for the Flow–Compaction Process of Thermosetting Epoxy Resin-Based Composites

**DOI:** 10.3390/polym17060722

**Published:** 2025-03-10

**Authors:** Ye Jing, Zhenyi Yuan, Kai He, Lingfei Kong, Guigeng Yang, Kaite Guo

**Affiliations:** 1 School of Mechanical and Precision Instrument Engineering, Xi’an University of Technology, Xi’an 710072, China; jy1339116830@gmail.com (Y.J.); 17391664669@163.com (K.H.); lingfeikong@xaut.edu.cn (L.K.); guigengyang@xaut.edu.cn (G.Y.); 2 School of Aerospace Science and Technology, Xidian University, Xi’an 710072, China; guokaite@xidian.edu.cn

**Keywords:** composite, flow–compaction, porosity, finite element method, curing process

## Abstract

Addressing the issue of porosity evolution during the curing process of thermosetting epoxy resin-based composites, a simulation model has been developed to describe the flow–compaction behavior of the composites aiming to predict changes in porosity throughout the curing process. Initially, a multi-physics coupling model encompassing sub-models for thermo-chemical, fiber bed compression, void compression, and percolation flow was established. This model accurately describes the changes in porosity within the composites during the flow–compaction process. The UMAT subroutine of the ABAQUS finite element analysis software was utilized to integrate these sub-models into the software. The validity of the simulation model was verified through corresponding experimental porosity measurements. The research further indicates that the porosity at the fillet of L-shaped composite components is higher than that in flat areas due to insufficient shear slip capacity. The results show that the porosity of the rounded corners of the L-shaped composite members is higher than that of the flat plate region due to the lack of shear slip capacity, and the fiber bed stiffness and inter-ply friction coefficient play an important role in the change in porosity.

## 1. Introduction

Thermosetting epoxy resin-based composites are increasingly being utilized across various industries, including aerospace, energy, automotive, and construction, owing to their exceptional properties such as high specific stiffness and strength, extended fatigue life, and excellent corrosion resistance [1]. Despite significant advancements in fabrication techniques, it remains nearly impossible to completely eliminate defects in composite parts. Among these defects, porosity is one of the most critical, primarily caused by entrapped air before curing (e.g., air retained within the raw prepreg material or introduced during the layup process) and volatiles generated during resin cross-linking. These voids severely compromise the mechanical properties of composites including inter-laminar shear strength, compression strength, transverse tensile strength, and fatigue life [2,3]. These properties can degrade by up to 10% for every 1% increase in void content [4]. Voids act as stress concentrators under loading conditions, accelerating crack initiation and propagation. Moreover, the presence of voids disrupts the stress transfer efficiency between fibers and the surrounding matrix, resulting in localized weaknesses that degrade the overall structural integrity.

Research on the formation and evolution of voids during composite manufacturing has a long and well-documented history. Chambers et al. [5] found that increasing the void content decreased the flexural strength and fatigue properties of the composites by varying the vacuum pressure during the curing process to make changes in the voids and using image analysis to characterize the voids. The curing process was modeled by Loos and Springer [6], who related the curing cycle to the thermal, chemical and physical processes that occur during curing. They explained that after voids are formed, their size may change due to thermal expansion, diffusion effects, or changes in void pressure due to changes in ambient temperature and pressure. Good agreement between experimentally measured void contents as a function of curing pressure and those predicted by Loos and Springer’s model has been found in the literature on hot press processing [7]. Wood and Bader [8] developed a diffusion model that can predict the rate of growth or collapse of entrapped voids in the resin, with the advantage of accounting for surface tension. They concluded that voids can collapse, and their growth can be suppressed by control of pressure and temperature even if the resin is saturated with a gas. Ledru et al. [9] proposed a coupled visco-mechanical and diffusion void growth model, which considers surface tension as well as pressure sensitivity. Through this model, it was concluded that there are three key parameters influencing the void size evolution: the onset of pressure application, the concentration of diffusive species, and the diffusion coefficient. Boey and Lye [10] showed that without the application of vacuum, void content can be reduced by increasing the cure pressure, but even with high pressures, a small degree of void is present. Olivier et al. [11] found the size, shape, and distributions of voids alter with cure cycle parameters. Lukaszewicz et al. [12] showed that high-quality carbon/epoxy laminates, with respect to void age, can be produced from autoclave prepregs without application of debulking and additional pressure, but with sufficient compaction of each ply during the automated laying process at elevated pressure and temperature.

The research conducted by the aforementioned scholars has demonstrated that the mechanisms underlying void formation are exceptionally intricate and contingent upon a multitude of factors including material properties, process parameters, and mold constraints, among others. Consequently, predicting the real-time distribution, size, shape, or morphology of voids presents a formidable challenge. Alternatively, by approaching the problem from the perspective of structural porosity evolution at the macroscale and obtaining porosity evolution under different pressures, temperatures, layer orientations, and thicknesses, we can explore and elucidate the relationships between relevant parameters such as pressure, temperature, and porosity evolution. This approach can circumvent the challenges posed by void geometric characteristics in real-time simulation predictions and experimental measurements. Ideally, by modeling the curing process for specific curvatures, one could evaluate the feasibility of a particular part geometry and layup sequence, and potentially refine or optimize the process parameters for manufacturing prior to actual part production.

Among these methods, the flow–compaction model is widely used. The underlying principle is the application of external pressure during the resin curing process, promoting uniform resin flow and expelling air bubbles and volatiles, thereby effectively reducing porosity. It is characterized by the complexity of multi-physics coupling, including thermochemical reactions, fiber bed compression, resin flow between fiber bundles, and bubble compression behavior. Many scholars have conducted research on flow–compaction. Loos and Springer [6] were among the first to propose a Sequential Compaction Model (SCM), assuming that the external pressure is solely borne by the resin. Gutowski proposed the Squeeze Sponge Model (SSM) [13,14], also known as the effective stress theory, assuming that the pressure is jointly borne by the resin and the fiber bed. Li [15] combined the finite element method with the SSM model to establish a two-dimensional numerical model to simulate the flow–compaction behavior of composite panels. Levy and Hubert [16] proposed a two-dimensional analytical model to predict the thickness deviations of L-shaped laminated plates with different mold radii and flange lengths. Hernández et al. [17] studied the effects of the material curing cycle on resin porosity, shape, and distribution. Sommi et al. [1] developed a multi-field coupling model, integrating heat conduction, flow–compaction, and resin rheology, to predict whether porosity might form during the molding process. Barari et al. [18] established a porosity evolution model considering the presence of initial voids, based on the ideal gas law and inter-fiber flow mechanisms. Blackwell et al. [19] further considered the impact of the nonlinearity of fiber bed thickness compression and successfully predicted fiber volume content and porosity. However, the viscosity due to variation in temperature and degree of cure was ignored.

A predictive model for porosity was formulated by Barari et al. [18]. This three-dimensional framework decomposes the behavior of prepreg materials into three distinct components that influence its stress response: the fiber bed, the hydrostatic compression of porosity within the resin, and the resin flow relative to the fiber bed. For each of these components, constitutive equations were derived, assuming a linearly elastic fiber bed, resin porosity governed by the ideal gas law, and Darcian flow characteristics. The model presented in this study builds substantially upon the methodology employed by Barari et al., incorporating several enhancements. Notably, it integrates real-time variations in temperature and cure degree into the model. Additionally, the formulation for the fiber bed has been extended to account for non-linear stiffening behavior, and the impact of inter-ply friction has been taken into consideration.

## 2. Mathematical Model

The flow–compaction stage of composite materials involves multiple physicochemical processes, including heat conduction associated with the exothermic resin curing reaction, resin flow between fiber bundles caused by viscosity changes, compression deformation of the fiber bed, and hydrostatic pressure variations due to initial internal porosity, among others. Furthermore, these physicochemical changes are coupled with one another. For example, variations in temperature and the degree of cure (DoC) result in alterations in resin viscosity, which subsequently alter the resin flow process between fiber bundles. Similarly, changes in resin content affect the stiffness of the fiber bed, hydrostatic pressure, and inter-bundle resin flow processes, among others.

During the flow compaction stage, the application of curing pressure induces volumetric and shape changes in the prepreg, accompanied by resin flow between fiber bundles. The prepreg, composed of fibers and resin, can be analyzed by decomposing the aforementioned phenomena into the contributions of the fiber bed and the resin in bearing the curing pressure. Furthermore, the mechanical response of the resin is subdivided into the compression of existing pores and the resin flow between fiber bundles. Consequently, the total curing pressure, *P*_total_, is supported by three sub-models: inter-bundle flow, fiber bed compression, and pore pressure, as expressed in Equation (1).

(1) $$P_{\mathrm{total}}=\sigma_{percolation}+\sigma_{fiberbed}+\sigma_{void}$$

where $\sigma_{percolation}$ represents the stress generated by the flow of resin between fiber bundles; $\sigma_{fiberbed}$ represents the fiber bed response; $\sigma_{void}$ represents the stress due to void compression during flow–compaction process.

**Figure 1:**
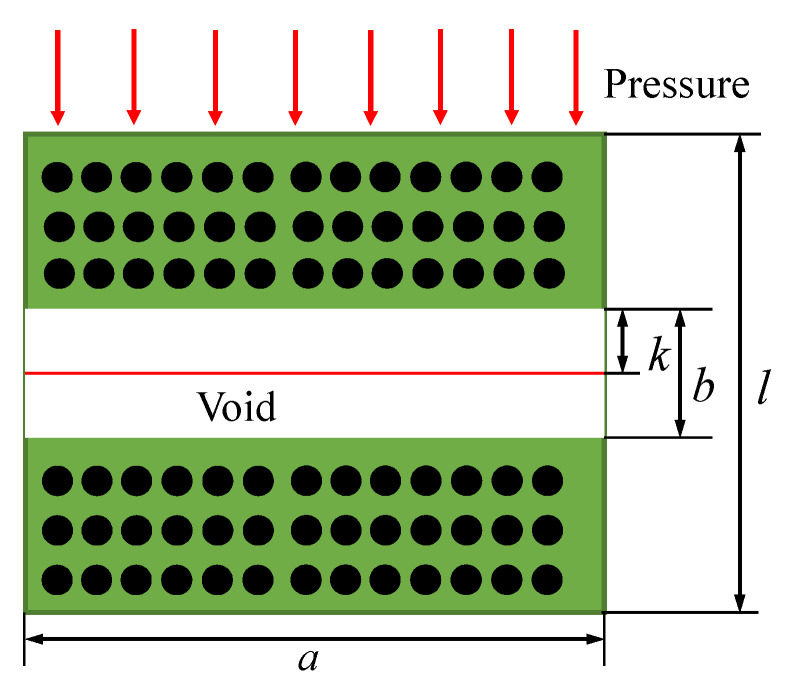
Diagram of void compression during flow–compaction process.

As the composite material preform undergoes compression during the flow–compaction process, compressive strain arises in the direction of thickness. This compressive strain is transmitted to the internal voids, leading to void compression and, consequently, a reduction in porosity. Assuming the composite material has dimensions of length × width × height as *a* × *c* × *l*, with a two-dimensional cross-section as illustrated in Figure 1, the initial fiber volume of the prepreg is *c* × *S*_1_. In the absence of voids, the fiber volume fraction is *V*_0_. During the lay-up process, an initial void with a height of *b* is introduced. The initial porosity volume fraction is *φ*_0_, and the fiber volume fraction before entering the autoclave for curing is *V_f_*_0_. The following relationships can be established:

(2) $$V_{0}=\frac{S_{1}\times c}{a\times \left(l-b\right)\times c}=\frac{S_{1}}{a\left(l-b\right)}$$

(3) $$V_{f0}=\frac{S_{1}\times c}{a\times l\times c}=\frac{S_{1}}{al}$$

By combining the above equations, we obtain:

(4) $$V_{f0}=V_{0}\left(1-\frac{b}{l}\right)=V_{0}\left(1-\varphi_{0}\right)$$

where

(5) $$\varphi_{0}=\frac{b}{l}$$

As the voids are compressed, their volume gradually decreases and may even dissipate, leading to an increase in the fiber volume fraction. Consequently, the real-time fiber volume fraction *V_f_* and the real-time porosity *φ* can be expressed as functions of the compression distance *k* in the thickness direction, as follows:

(6) $$V_{f}=\frac{S_{1}\times c}{a\times \left(l-k\right)\times c}=\frac{S_{1}}{a\left(l-k\right)}$$

(7) $$\varphi =\frac{a\times \left(b-k\right)\times c}{a\times l\times c}=\frac{b-k}{l}$$

In combination with Equations (4) and (5), we obtain:

(8) $$V_{f}=\frac{V_{f0}}{1-k/l}=\frac{V_{f0}}{1+\varepsilon_{33}}$$

(9) $$\varphi =\varphi_{0}+\varepsilon_{33}$$

where *ε*_33_ represents the compressive strain in the thickness direction of the composite material, with a negative value taken in the equation; *φ*_0_ denotes the initial porosity of the composite material, which can be obtained using experimental measurement. *V*_0_ can be obtained from the material specifications provided by the supplier.

### 2.1. Thermo-Chemical Model

The thermo-chemical model performs a decoupled calculation of the internal temperature and DoC during the curing process. The computed temperature and DoC are subsequently utilized to ascertain the viscosity and hydrostatic pressure of the resin. During the heat transfer process, it is imperative to consider convective heat exchange between the high-temperature gas within the autoclave and the composite material at the external boundary. Within the material, the heat generated by the resin cross-linking reaction is considered, as expressed in Equation (10) [20]:

(10) $$\lambda_{x}\frac{{𝜕}^{2}T}{𝜕x^{2}}+\lambda_{y}\frac{{𝜕}^{2}T}{𝜕y^{2}}+\lambda_{z}\frac{{𝜕}^{2}T}{𝜕z^{2}}+\rho_{r}(1-V_{f})H_{r}\frac{d\alpha }{dt}=\rho_{c}C_{c}\frac{𝜕T}{𝜕t}$$

where *ρ*, *C* and *λ* represent the material density, specific heat capacity, and thermal conductivity, respectively. *x*, *y* and *z* denote the three directions of an anisotropic material. The subscripts *r*, *f* and *c* represent the resin, fiber, and composite material, respectively. *T* and *t* represent temperature and time, respectively. *V_f_* is the fiber volume fraction. *H_r_* is the total heat released per unit mass of resin during the curing reaction. *α* and d*α*/d*t* represent the degree of cure and the curing reaction rate, respectively. For example, for AC531 resin, this can be expressed as:

(11) $$\frac{d\alpha }{dt}=K_{1}\alpha^{m}{\left(1-\alpha \right)}^{n_{1}}+K_{2}{\left(1-\alpha \right)}^{n_{2}}$$

where

(12) $$K_{i}=A_{i}\exp(\frac{-\Delta E_{i}}{RT})$$

where *K_i_* represents the reaction rate constant. *A_i_* is the pre-exponential factor. △*E_i_* is the activation energy. *R* is the ideal gas constant. *m*, *n*_1_ and *n*_2_ are fitting constants, with specific values provided in Table 1.

### 2.2. Percolation Flow Model

The resin flow process modifies the distribution of resin content within the structure. It is commonly presumed that the resin behaves as an incompressible material under external loads, whereas the internal pores exhibit compressibility. The percolation flow process is driven by resin pressure, enabling the transfer of resin within the prepreg. This percolation flow behavior of the resin between fiber bundles can also generate stress within the prepreg, counteracting part of the external load. It is noteworthy that, due to the viscoelastic properties of the resin, the stress induced by percolation flow will progressively diminish or potentially vanish over sufficiently extended time scales. Based on Darcy’s law, the stress induced by the flow of a viscous fluid in a porous medium with a certain permeability is given by the following equation:

(13) $$\sigma_{percolation}=\frac{\eta D^{2}}{K}\dot{\varepsilon }$$

where *η* represents the resin viscosity, with the viscosity of AC531 resin detailed in Reference; $\dot{\varepsilon }$ denotes the strain rate during the compaction stage; *D* is the characteristic length between internal pores; *K* is the fiber permeability. The fiber permeability in the thickness direction can be expressed as a function of the fiber volume fraction, as shown in Equation (14) [21]:

(14) $$K=c_{2}r_{f}^{2}{\left(\sqrt{V_{f}^{\max}/V_{f}}-1\right)}^{5/2}$$

where *r_f_* represents the fiber radius, which is taken as 0.004 mm in this context. $V_{f}^{\max}$ denotes the maximum achievable fiber volume fraction. For in-plane fiber distribution patterns such as triangular, quadrilateral, pentagonal, or hexagonal arrangements, its value ranges from 0.60 to 0.91. Here, the average value of 0.78 for these four cases is adopted. *c*_2_ is a fitting constant, which is also related to the fiber arrangement pattern, with values ranging from 0.69 to 0.23 [21].

### 2.3. Fiber Bed Compression Model

Under external loading, the fiber bed undergoes deformation as well, with the magnitude of deformation exhibiting significant variation across the three principal directions owing to the anisotropic nature of the fiber bed. It is generally assumed that the elastic modulus *E*_1_ in the fiber direction remains constant during the curing and compaction process, while the in-plane elastic modulus *E*_2_, perpendicular to the fiber direction, is much smaller and significantly lower than *E*_1_. The stress in the thickness direction under load is more complex and is related to the fiber volume fraction and the strain in the thickness direction, as expressed below [14]:

(15) $$\sigma_{fiberbed}=A_{s}\frac{\sqrt{V_{f}/V_{0}}-1}{{\left(\sqrt{V_{f}^{\max}/V_{f}}-1\right)}^{4}}$$

where *A_s_* represents the stiffness fitting coefficient of the fiber bed, which characterizes the compressive resistance of the fiber bed. In this case, it is taken as 500 Pa. Combining with Equation (8), Equation (15) can be rewritten as:

(16) $$\sigma_{fiberbed}=A_{s}\frac{\sqrt{1/1+\varepsilon_{33}}-1}{{\left(\sqrt{V_{f}^{\max}/V_{f0}}\sqrt{1+\varepsilon_{33}}-1\right)}^{4}}$$

### 2.4. Void Pressure Model

The voids embedded within the structure are encircled by resin, leading to a plausible hypothesis that the pressure external to these voids is equivalent to the pressure of the surrounding resin. In such a scenario, the voids exhibit limited mobility relative to the fiber matrix. When the voids are subjected to compression, an opposing reactive force is generated to counteract the external load. This force can be referenced and understood through the application of the ideal gas state equation. This resistance force is related to both pressure and temperature. As the external resin pressure on the voids increases, the voids will gradually compress and shrink, or even be eliminated. To streamline the model, it is assumed that the initial voids are uniformly distributed throughout the structure. The hydrostatic pressure generated by void compression within the resin is given by the following equation [18]:

(17) $$\sigma_{void}=\frac{P_{0}\varphi_{0}}{\varphi_{0}+\varepsilon }\left(\frac{T}{T_{0}}\right)\left(1-d_{v}\right)-P_{0}$$

where

(18) $$d_{v}=\sigma_{void}\left(\frac{H\rho_{r}}{\varphi_{0}\rho_{g}}\right)\left(1-\varphi_{0}-\varepsilon -\frac{V_{0}}{1+\varepsilon }\right)$$

where *T*_0_ represents the initial temperature; *φ*_0_ is the initial porosity; *P*_0_ represents the initial pressure of the voids, which is commonly presumed to equate to atmospheric pressure, 0.1 MPa; *H* is Henry’s constant, used to determine the solubility of gas in liquid, and can be obtained through experimental measurement; *ρ*_g_ is the density of volatile substances, which can be taken as 1.0 kg/m^3^; *d_v_* represents the proportion of the original volatile substances removed from the voids during the compaction flow stage. This value is very small, so it can be neglected and simplified in the model. Therefore, Equation (17) can be simplified as:

(19) $$\sigma_{void}=\frac{P_{0}\varphi_{0}}{\varphi_{0}+\varepsilon }\left(\frac{T}{T_{0}}\right)-P_{0}$$

### 2.5. Numerical Implementation

**Figure 2:**
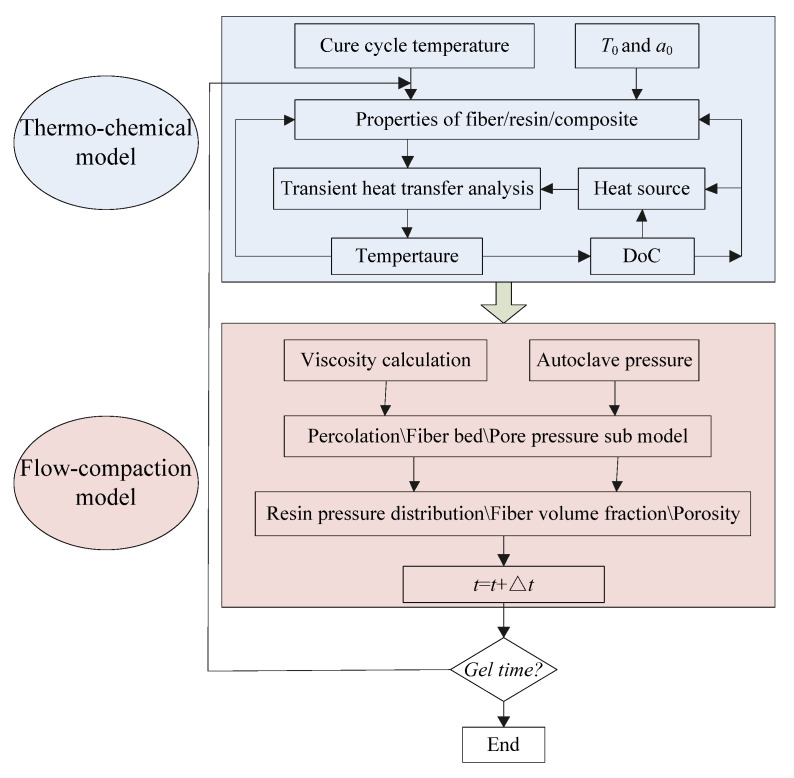
The flowchart of flow–compaction model.

Subsequently, the aforementioned thermo-chemical and flow–compaction models have been incorporated into a simulation framework, as illustrated in Figure 2. Initially, the baseline material properties serve as inputs for solving the heat transfer and curing kinetic equations within the thermo-chemical model, yielding temperature and DoC distributions. These outputs are subsequently employed to update the time-varying parameters pertaining to the rheological and mechanical properties of the fibers, resin, and the composite material as a whole. After computing the rheological parameters, such as the viscosity of the resin and the permeability of the fibers, the dynamics of resin flow, the compression of the fiber bed, and the pressure exerted on void compression are analyzed by utilizing Darcy’s law and the theory of effective stress. Subsequently, the resin pressure is revised, resulting in a reconfigured distribution of fiber volume fraction and porosity. This iterative procedure continues until the resin reaches the gel point. By employing this multi-physics coupled finite element (FE) model, the development of temperature, DoC, fiber volume fraction, and non-uniform porosity during the curing process can be calculated.

In this methodology, the governing equations and models are implemented within the ABAQUS software framework for the purpose of simulating the flow-induced compaction characteristics of an L-shaped laminated composite material. A FORTRAN subroutine is utilized to delineate material properties, boundary conditions, loading scenarios, and additional parameters, thereby substantially augmenting the capabilities and versatility of the software. The user subroutine UMAT (which stands for user material subroutine for defining a material’s mechanical behavior) empowers users to tailor the constitutive equations of materials, which are often not pre-installed or readily available within the software. The subroutine also provides the user with the capability to integrate, modify, and archive any state variables pertinent to the implemented material model.

**Table 1:** Properties for AC531 resin and CCF800 fiber.

Parameters	Symbol	Value	Resource
Resin density	*ρ*_r_/(kg·m^−3^)	1300	[22]
Composite specific heat	C_c_/(J·(kg·K)^−1^)	1312	[23]
Composite density	*ρ*_c_/(kg·m^−3^)	1540	
Composite conductivity in the longitudinal direction	λ_c_^L^/(W·(m·K)^−1^)	3.154	
Composite conductivity in the transverse direction	λ_c_^T^/(W·(m·K)^−1^)	0.159	
Frequency factor of autocatalysis model	*A*_1_/s^−1^	3384	
Frequency factor of autocatalysis model	*A*_2_/s^−1^	7,536,410	
Fitting constant	*m*	0.6062	
Fitting constant	*n*_1_	3.6286	
Fitting constant	*n*_2_	0.8712	
Activity energy of autocatalysis model	△*E*_1_/(J·mol^−1^)	55,575	
Activity energy of autocatalysis model	△*E*_2_/(J·mol^−1^)	94,596	
Total heat energy released by curing	*H_r_*/(J/kg)	277,733	
Fiber modules	*E*_1_/GPa	147,000	[24]
Fiber bed transverse modulus	*E*_2_/MPa	9.25	[24]
Voids distance	*D*/mm	0.1	[19]

## 3. Experimental Processes

**Figure 3:**
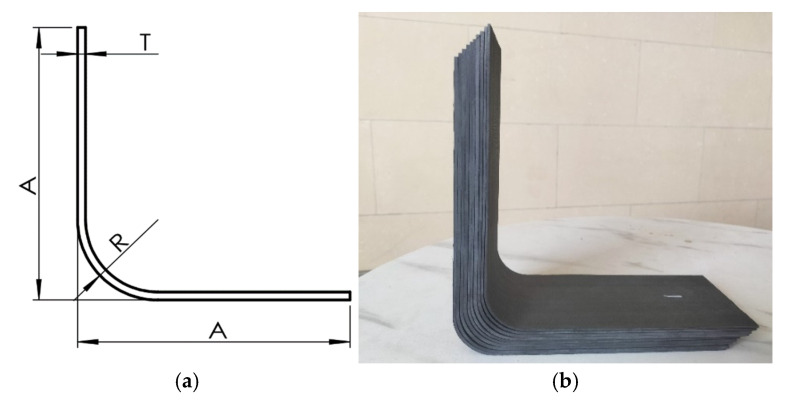
Preparation of L-shaped composite material parts. (**a**) Dimensions of L-shaped composite material parts; (**b**) cured L-shaped composite material parts.

The composite prepreg used in this study is AC531/CCF800H manufactured by China Aviation Composite Materials Company, Beijing, China, with a single-layer prepreg thickness of 0.180 mm and an initial fiber volume fraction of 65%. The molding process inside the autoclave is as follows: the heating rate is 2 °C/min, reaching 190 °C, followed by a 3 h dwell time at 190 °C. The cooling rate is 3 °C/min until the temperature reaches 65 °C, at which point the part is removed from the autoclave. During the process, no pressure is applied before reaching 60 °C, and only the vacuum bag pressure is used. After 60 °C, a curing pressure of 0.5 MPa is applied until the composite is removed from the autoclave. The part dimensions and the cured part are shown in Figure 3, where A is 200 mm, R is 30 mm, and the thickness T varies with different layup angles.

**Figure 4:**
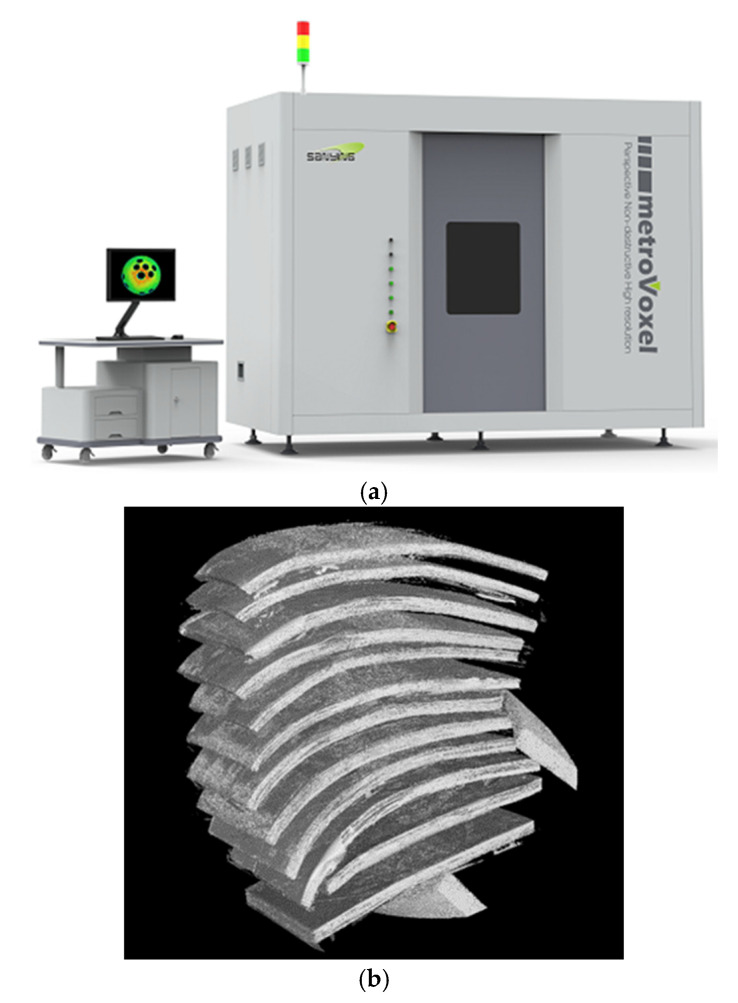
CT scanner and model reconstruction of composite l-shaped parts. (**a**) Nano Voxel computed tomography (CT) scanner; (**b**) the reconstructed model based on CT scan three-dimensional data.

The L-shaped component undergoes a vacuum bagging process during the lay-up and curing procedure. After the prepreg is fully laid up, the part is first subjected to computerized tomography (CT) scanning. Once the curing process is completed, the part is removed from the mold and scanned once more to ascertain the porosity levels before and after the curing stage. In this experiment, the CT nanoVoxel-2000 supplied by Sanying Precision Instruments Co. Ltd., Tianjian, China, is used for measuring the porosity of the model. The reconstructed model based on CT scan three-dimensional data Figure 4a. During the CT scanning process, an X-ray source emits X-rays towards the composite material. When X-rays traverse a material, they undergo a decrease in energy due to absorption by the material itself. The information pertaining to these absorption and scattering phenomena, particularly the direction and intensity of the scattered rays, offers valuable insights into the internal structure of the material. This includes details about the size, shape, and distribution of pores within the material. The X-ray information, which consists of X-rays that have been attenuated by the material, is received by a detector and converted into electrical signals. These signals are then transmitted to a computer for further processing. By utilizing the collected data and employing specific reconstruction algorithms, such as the filtered back-projection algorithm, the computer is able to generate a three-dimensional image that accurately depicts the internal structure of the composite material. This image clearly displays the pores, inclusions, cracks, and other defects within the material. By analyzing the reconstructed images, the porosity within the composite material can be accurately measured, which is defined as the percentage of pore volume relative to the total volume. The CT scan results are processed using Avizo software 2019.1, and the three-dimensional reconstruction model is shown in Figure 4b.

**Figure 5:**
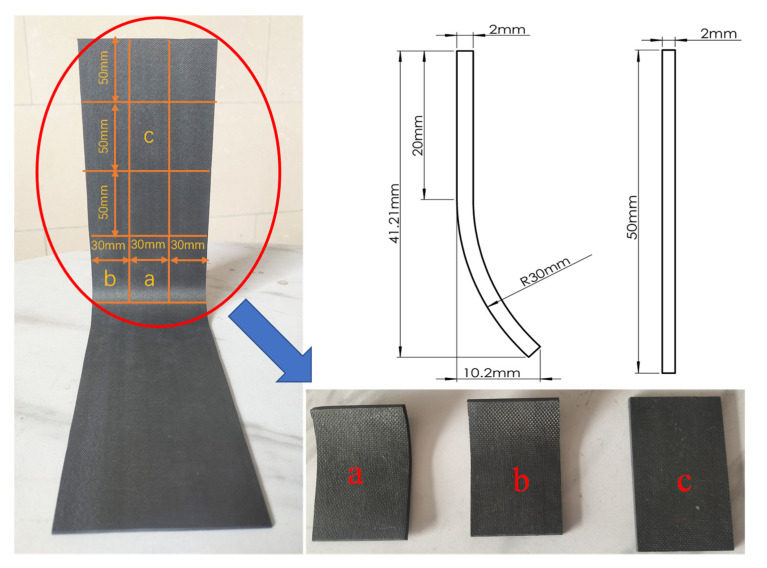
Schematic diagram for cutting [0/45/−45/90]_2s_ laminate composite material parts.

**Figure 6:**
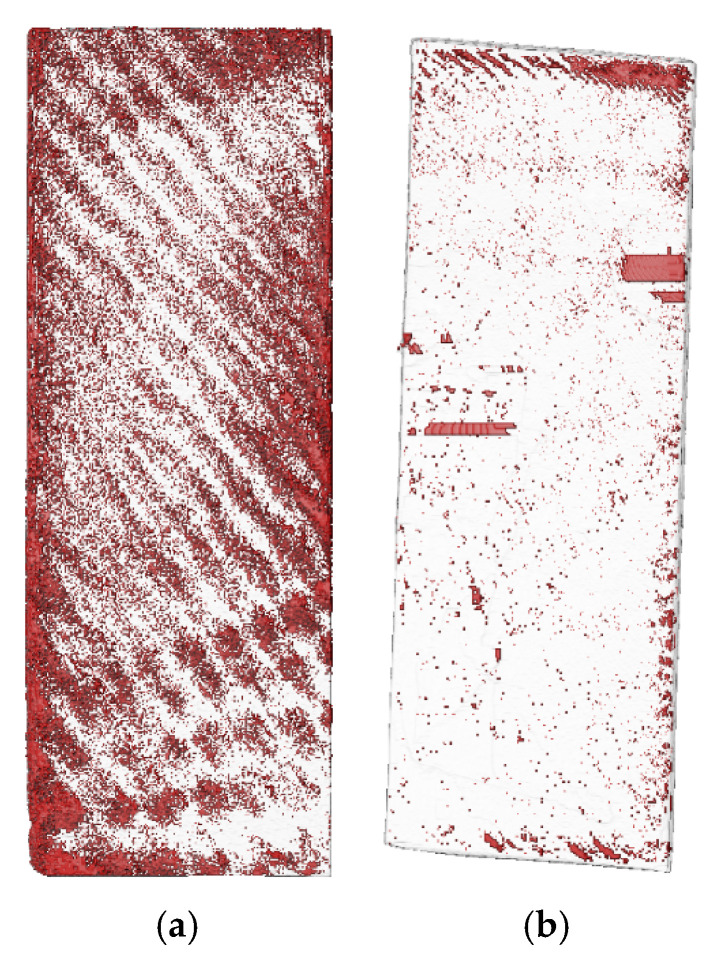
Scanning results of void content in [0/45/−45/90]_2s_ composite material component (red represents voids, and white represents areas without voids). (**a**) Before curing; (**b**) after curing.

The industrial CT scanner utilized in this experiment possesses a scanning capability of up to 1 m within the plane, with a recommended maximum dimension of 100 mm within the same plane. On one hand, the larger the size of the part, the worse the recognition of porosity, as the part’s overall dimensions increase. On the other hand, in order to obtain multiple sets of experimental data for the prepreg in one CT scan, the parts were cut after the lay-up and curing process. Taking the L-shaped component with a [0/45/−45/90]_2s_ (the subscript 2 represents that the ply sequence is repeated twice, and subscript s stands for symmetric ply. Therefore, [0/45/−45/90]_2s_ represents [0/45/−45/90/0/45/−45/90/90/−45/45/0/90/−45/45/0]) lay-up as an example, four parts were laid up simultaneously during the layup process. Two of the prepreg parts were scanned after the vacuum bagging process, and their porosity was measured before curing. The other two prepreg parts underwent further curing in the autoclave, and after curing, CT scans were conducted to measure the porosity of the finished parts. The cutting dimensions of the parts are shown in Figure 5. By comparing the cut pieces at positions a and b, it was verified that the internal porosity of the parts is independent of the width of the laid-up parts. Additionally, comparing positions a, b, and c, the porosity of the straight-edge regions was found to be independent of the dimensions of the straight-edge sections. Both pre-cured and cured measured parts were cut, and the cut test pieces at positions a and b included porosity data for both the rounded and straight-edge regions, while position c only provided the porosity scan results for the straight-edge region. The porosity in the flat and rounded regions of the sample was identified separately, and the post-CT scan rendered images are shown in Figure 6. The measurement results are listed in Table 2.

From the data in Table 2, it can be seen that the porosity results for the four samples at regions a and b, whether at the rounded corner or straight-edge positions, are essentially the same when comparing pre-cured samples 1 and 2, or comparing cured samples 3 and 4. The porosity results for samples 1 and 2, or 3 and 4, are very consistent, indicating that in the current sample dimensions, porosity is independent of the width direction size. This suggests that cutting small test pieces can be used for porosity measurements. Furthermore, for all four samples, the porosity results at straight-edge regions a, b, and c are also very similar, showing that the cutting test piece method can also be applied for porosity measurement along the straight-edge direction.

Table 2 also shows that for pre-cured samples 1 and 2, the porosity at the six flat regions is consistent, with an average of 6.59%. The porosity at the four corner regions is also consistent, with an average of 7.96%. For cured samples 3 and 4, the porosity at the six flat regions is similarly consistent, with an average of 2.02%, and the porosity at the four corner regions is also consistent, with an average of 2.94%. This indicates that the quality control during the lay-up process was good. The porosity levels observed at the rounded corner positions are greater compared to those at the flat regions. This is partially attributed to the fact that the pressure transfer effect is less effective at the rounded corners compared to the flat areas. On the other hand, the interlayer slippage at the rounded corner is constrained by the adjacent flat regions, which limits the slippage ability during pressure application. As a result, the compression of the initial air bubbles is insufficient at the rounded corner, leading to higher porosity in these areas compared to the flat regions. It should be noted that the straight-edge length of the L-shaped part in this study is 200 mm. If the thickness of the part increases, further research is needed to determine whether the porosity at in-plane positions will remain consistent.

## 4. Discussion

### 4.1. Finite Element Analysis

**Figure 7:**
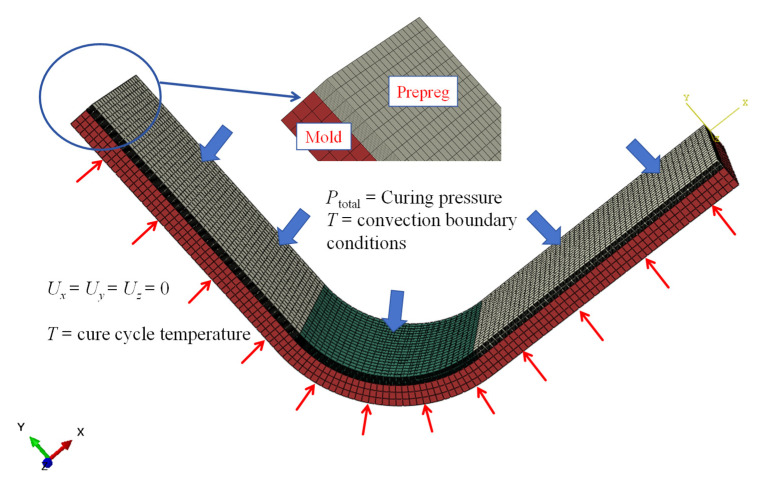
Applied boundary conditions of the FE model.

A computational analysis is performed utilizing the proposed flow–compaction simulation approach within the commercial software package ABAQUS 2021. The L-shaped laminate is modeled based on the dimensions of the experimental specimens. In the finite element (FE) model, auxiliary elements such as vacuum bags and breathable materials are excluded, with their influences being accounted for by the application of pertinent loads and boundary conditions. To eliminate any mesh density dependency, an initial assessment is conducted to examine the influence of mesh density on the predicted outcomes. One mesh was set for each layer through the thickness direction of the part, and both the mold and the composite material were modeled using C3D8 elements. For parts with layups excluding ±45°, a quarter-model approach was used to decrease the element count and enhance computational performance. The boundary conditions and meshing of a quasi-isotropic stacking sequence [0/45/−45/90]_2s_ are shown in Figure 7. The outer surface of the laminate exposed to the gas in the autoclave is set with convective heat transfer boundary conditions and curing pressure. Due to the high thermal conductivity of the metal mold, the bottom surface of the mold is applied at the temperature defined by the cure profile. All other boundaries are set to be adiabatic. The coefficient of friction was set to 0.2 for both the interlayer interactions within the composite material and the interface between the mold and the composite material [25,26]. The properties of the composite used in the FE model are listed in Table 1.

**Figure 8:**
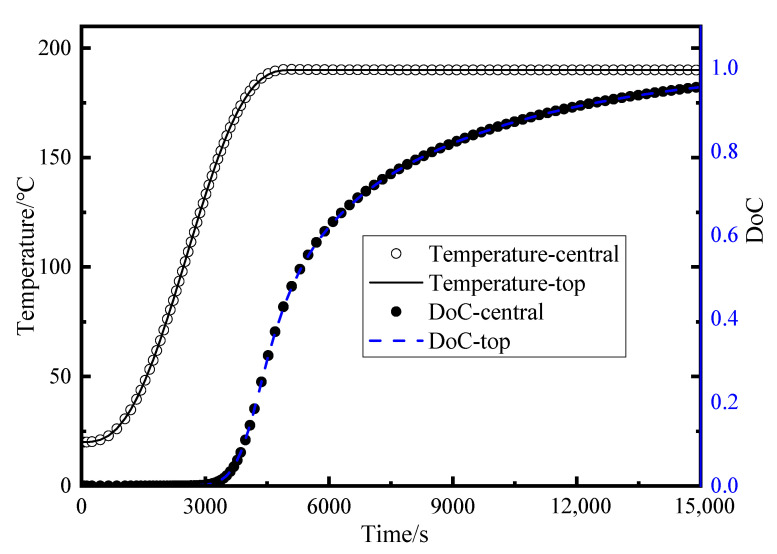
The simulation results of temperature and DoC at the surface and center point of the composite part.

**Figure 9:**
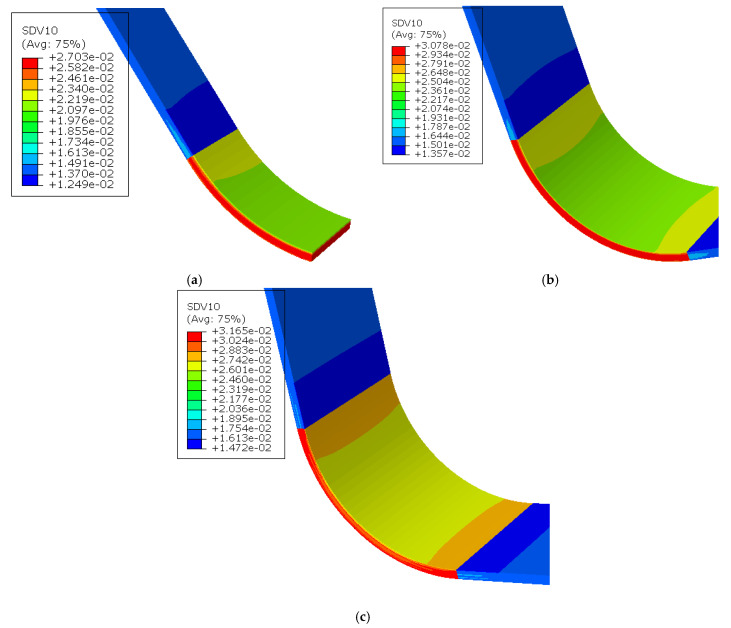
Updated porosity content distribution for three different laminates (SDV 10 represents the porosity of the composite). (**a**) The [0]_12_ layup; (**b**) [0]_16_ layup; (**c**) [0/45/−45/90]_2s_ layup.

The outcomes of the thermal–chemical analysis present the variations in temperature and DoC at the central node and the top surface of the laminate, as depicted in Figure 8. It is noteworthy that both temperature and DoC exhibit a relatively uniform distribution across the thickness of the composite laminate. This uniformity arises due to the laminate’s relatively thin thickness, which facilitates prompt heat transfer from the exterior and internal exothermic reactions in the direction of the thickness. Subsequently, the viscosity and void pressure within the flow–compaction model are updated using the instantaneous results derived from the temperature and DoC inputs. Upon completion of the simulation model, a comparison was conducted between the average porosity at the designated experimental locations and the experimental findings. The simulation outcomes for three distinct layup configurations are presented in Table 3, Figure 8 and Figure 9. It is evident that the simulation results align well with the experimental data, with a maximum error of 15.3% in the flat region and 13.5% in the corner region. This demonstrates that the established model has high accuracy.

Generally, the experimental results align with the anticipated pattern, wherein the corner sections exhibit a higher final porosity compared to the flat sections in unidirectional layups and in cross-ply parts, as shown in Table 3. The plies at the rounded corner experience resistance from the adjacent straight edge sections during relative slippage, resulting in less compressive deformation in the thickness direction and higher porosity. It can be observed that for unidirectional 0-degree plies, the porosity of 16 plies is greater than that of 14 plies. This indicates that as the stiffness of the part increases, its ability to resist deformation in the thickness direction is enhanced, and the relative slippage between plies becomes less prone. Therefore, under the same ply configuration, an increase in thickness leads to an increase in porosity. A nesting effect can occur between the fibers in 0-degree plies (this content will be discussed in Section 4.2), allowing the fiber bed to become more densely packed after compaction. Therefore, the porosity of a pure 0-degree ply layup is lower than that of a cross-ply layup at the same thickness. This also indicates that the ply sequence significantly affects the compaction of the composite part.

### 4.2. Influence of Fiber Bed Stiffness on Porosity

**Figure 10:**
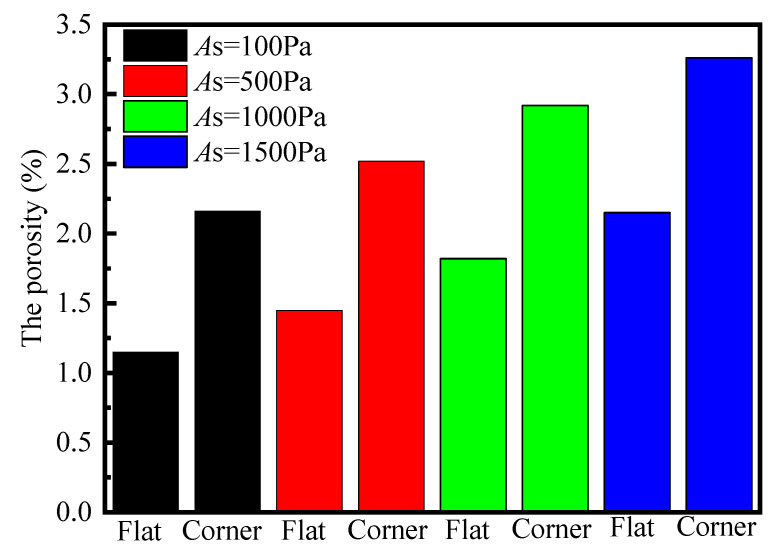
The effect of compaction curve scaling parameter on the final porosity of composite.

The fiber bed stiffness coefficient characterizes its ability to resist compression under pressure. Taking the [0]_12_ layup in the experiment as an example, with an initial fiber volume fraction of 65% and a final fiber volume fraction of 78%, while keeping other parameters constant and adjusting only the fiber bed stiffness. Different fiber bed stiffness parameters were set as *A*_s_ = 100 Pa, 500 Pa, 1000 Pa, and 1500 Pa to represent the ease or difficulty of compressing the fiber bed. The calculated results for the model’s porosity are shown in Figure 10. As can be seen, with an increase in fiber bed stiffness, the ability to resist external pressure improves, making the composite material more difficult to compress during the flow–compaction process. The pressure borne by the fiber bed increases, the hydrostatic pressure on the pores decreases and the porosity is reduced. Therefore, the final porosity increases as the fiber bed stiffness increases.

**Figure 11:**
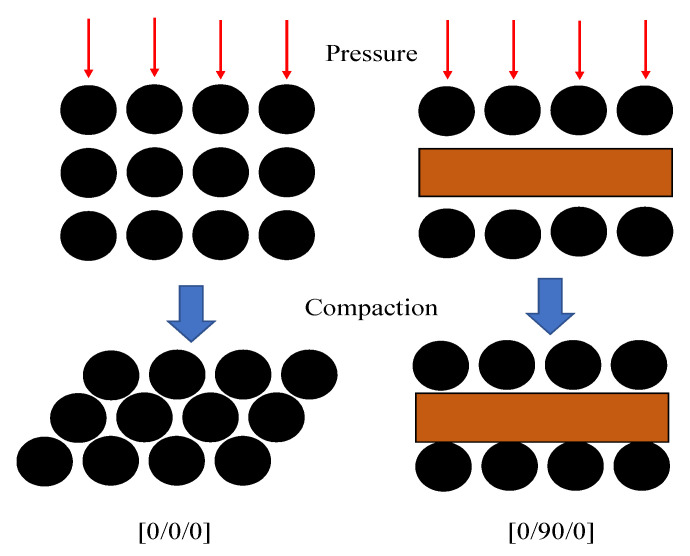
Nesting phenomenon during fiber compression.

It should be noted that different prepregs will exhibit varying compaction behaviors due to differences in fiber types, fiber arrangement patterns, and the degree of fiber interlocking (such as whether a 0° ply is adjacent to another 0° ply or to a 90° ply). A pure 0° layup exhibits a higher degree of fiber interlocking, and the overall fiber bed demonstrates relatively weaker stiffness, while alternating layups show stronger compaction resistance, as shown in Figure 11. As can also be seen in Table 3, whether the porosity measurement results are taken after initial vacuum pre-compaction or after curing, for the same initial thickness, the porosity of the [0/45/−45/90]_2s_ layup is higher than the porosity of the layup [0]_16_. This highlights that for structural components with stringent thickness requirements, the design of the layup sequence is crucial for thickness control. In subsequent model improvements, the fiber bed stiffness parameters will be obtained through compression experiments of prepregs designed according to different layup configurations.

### 4.3. Influence of Inter-Ply Friction on Porosity

**Figure 12:**
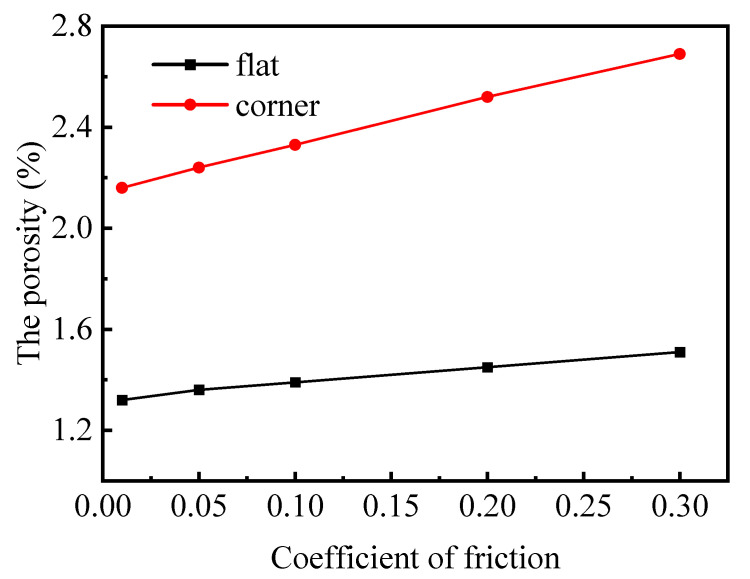
The effect of inter-ply friction on the final porosity of composite.

The coefficient of inter-ply friction also influences the compaction of porosity. A reduction in the coefficient of friction enhances the ability of the plies to slide relative to each other under shear forces. This decrease in friction weakens the resistance to compression between the plies, ultimately facilitating improved compaction. Taking the [0]_12_ layup in the experiment as an example, with an initial fiber volume fraction of 65% and a final fiber volume fraction of 78%, and keeping all other parameters constant, the coefficient of inter-ply friction was varied as 0.01, 0.05, 0.1, 0.2, and 0.3. The results are shown in Figure 12. It is observable that as the coefficient of inter-ply friction diminishes, the porosity levels in both the corner and flat regions decrease. Notably, the corner region demonstrates a greater sensitivity to variations in the coefficient of inter-ply friction compared to the flat region. In the case of thermosetting composites, as the resin undergoes a cross-linking reaction with increasing temperature, its viscosity decreases. This change has a notable impact on the inter-ply friction coefficient. Lower viscosity leads to stronger inter-ply sliding capability. However, after the resin gels, its viscosity sharply increases, severely limiting the inter-ply shear sliding ability. Therefore, it is critical to choose the appropriate timing for applying pressure in the actual process design.

### 4.4. Influence of Void Distance on Porosity

**Figure 13:**
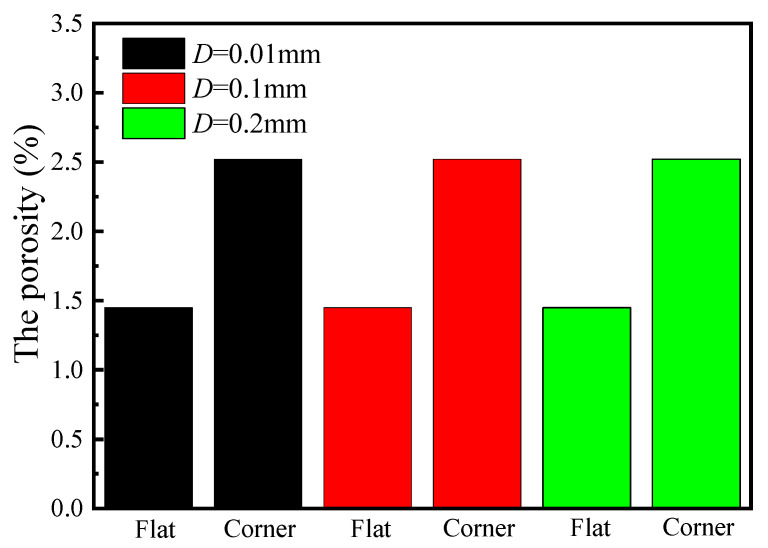
The effect of void distance on the final porosity of composite.

In the model, certain parameters such as the void distance *D* are challenging to measure directly. Typically, this value ranges from 0.01 mm to 1 mm. Taking the [0]_12_ layup from the experiment as an example, with an initial fiber volume fraction of 65% and a final fiber volume fraction of 78%, while keeping all other parameters constant, *D* values of 0.01 mm, 0.1 mm, and 0.2 mm were studied to investigate their influence on the final porosity. The simulation results are shown in Figure 13. It can be observed that void distance has no impact on the final porosity. This occurs because, on one hand, the stress generated by percolation flow is relatively modest, primarily serving to propel the resin from resin-rich areas towards resin-deficient regions. This process allows existing pores to be encapsulated by the resin, which then undergoes compression under the hydrostatic pressure exerted by the resin. Conversely, the resin demonstrates purely viscous behavior, meaning that over an extended duration, the stress generated by viscous flow will progressively diminish. Therefore, void distance does not influence the final porosity during the flow–compaction process.

## 5. Conclusions

(1) A three-dimensional finite element model describing the flow–compaction process of thermosetting resin-based composites was established based on four sub-models: thermochemical, percolation flow, fiber bed compression, and void pressure. Compared with existing research, this study simultaneously considers the effects of inter-ply slippage, changes in resin viscosity, and the compression of existing voids on the flow–compaction process of composite materials. Validation against experimental data demonstrates that the proposed model exhibits high accuracy.

(2) For L-shaped components, the reduced interlaminar sliding capability at corner regions leads to higher porosity compared to the straight-edge sections. During the curing process, pores are compressed, and the porosity gradually decreases.

(3) As the stiffness of the fiber bed increases, the pressure borne by the fiber bed rises, resulting in a reduction in hydrostatic pressure acting on the pores, thereby decreasing the degree of pore compression.

(4) As the friction coefficient decreases, the porosity in both corner and flat regions decreases, with the porosity at corner regions being more sensitive to changes in the friction coefficient compared to flat regions.

## Figures and Tables

**Table 2:** Experimental results of porosity (%) in [0/45/−45/90]_2s_ composite parts.

	Region	a1	b1	a2	b2	c1	c2
Before cure	Flat/%	6.78	6.21	6.85	6.47	6.42	6.83
	Corner/%	8.31	8.14	7.76	7.62		
	Region	a3	b3	c3	a4	b4	c4
After cure	Flat/%	2.11	1.99	2.24	1.88	1.82	2.06
	Corner/%	2.75	2.88	/	2.99	3.12	/

Where a, b, and c represent the three regions in which the specimen is cut; numbers 1 and 2 represent preforms 1 and 2; numbers 3 and 4 represent the cured parts 3 and 4.

**Table 3:** Experimental and simulation results of porosity (%) in L-shaped composite material parts.

Layup	Exp-Before Cure/%		Exp-After Cure/%		FEM-After Cure/%	
	Flat	Corner	Flat	Corner	Flat	Corner
[0]_12_	5.90	6.98	1.57	2.22	1.45	2.52
[0]_16_	6.28	7.57	1.73	2.75	1.61	2.93
[0/45/−45/90]_2s_	6.59	7.96	2.02	2.94	1.71	3.02

## Data Availability

The original contributions presented in this study are included in the article. Further inquiries can be directed to the corresponding author. Data will be made available on request.
